# Analysis of fatal congenital anomalies among pregnant women at Saudi Arabia using ultrasonography

**DOI:** 10.6026/973206300211176

**Published:** 2025-05-31

**Authors:** Nagla Hussein Mohamed Khalid, Norah Saeedan Alyami, Rahaf Saad Althobaiti, Naseem Ali Salem Al Salem, Sarah Saad Rashed Al Qatran, Ghaida Ali Alosaimi

**Affiliations:** 1 Department of Radiological Science, Najran University, Kingdom of Saudi Arabia

**Keywords:** Ultrasound examinations, congenital anomalies, pregnant women, gestational age, maternal pathology

## Abstract

The association between pregnant wome's health and the presence of congenital anomalies at Saudi Arabia is of interest. The total sample comprised of 100 individuals and the examinations took place in the period January 2023-January 2024. Relationships between health factors, demographic variables and foetal anomalies were assessed using Chi-square tests. With the majority of the participants being in the optimum childbearing age range, the average point of gestation at the time of the ultrasound assessment was 26.5 weeks and the average age of the women was 29.8-years old. Five major forms of congenital anomaly were observed among the 100 women examined. These were as follows: cardiac anomalies (34% of the total number of anomalies); musculoskeletal anomalies (22% of the total); central nervous system anomalies (20% of the total); gastrointestinal anomalies (15% of the total); and genitourinary anomalies (9% of the total), Just under a third (30%) of the sample reported that they had some form of adverse health condition (40% of this group had diabetes mellitus and 27% had hypertension).

## Background:

When anomalies (whether just a single one or multiple anomalies) are identified, either after birth or during gestation and these occur in the morphogenesis of organs (or body areas), these are referred to as congenital malformations [1]. Infant death, chronic disease and chronic disabilities can result from congenital abnormalities and they are a major issue for global and national health policymakers. When intrinsic anomalies occur during development, or when embryogenesis does not follow a normal path, there can be major deleterious effects on children, their families, the healthcare systems that provide care for them and the public institutions that support them, with life-long disabilities often ensuing. Neonatal and infant mortality and illness resulting from congenital malformations are critical issues in public health policy. Around 2-3% of individuals are born with congenital malformations globally, though there is some variation from region to region [2]. Around 3.2 million children experience physical and/or mental disabilities as a result of congenital malformations and 3.3 million children with such malformations die before the age of five, with a total of eight million born per annum with such defects [3]. The successful development of the foetus, the health of the mother and the on-going well-being of the child, are all significantly hindered by the occurrence of congenital anomalies, with a significant proportion of infant deaths and illnesses being accounted for by congenital anomalies. Potentially, by identifying wome's health issues and gauging the level of risk of foetal anomalies occurring, patient outcomes can be enhanced because mothers can be referred to appropriate specialists and treatment options in the prenatal phase [4-5]. Serving to empower parents by preparing them to face potential issues and enabling them to cope better with challenges, ultrasound examinations are often used to identify congenital anomalies during gestation and this facilitates the development of effective treatments and care plans [6]. There is some evidence of the fact that congenital anomalies are relatively highly prevalent in the region of Najran in Saudi Arabia, yet to date there is a dearth of data pertaining to how ultrasound examinations are used to identify foetal anomalies in Najran and how effective these examinations are in shaping health outcomes.

Collecting data on this in Najran will make a valuable contribution to the development of policy in the region, with the western part of Najran being particularly exposed to risk of congenital anomalies due to the fact that infection levels are higher (partly because of migration across the Yemen-Saudi Arabia border) [7-8]. Studies of the prevalence of congenital anomalies in the Saudi population at large have found relatively similar rates, with a recent study finding that the anomaly frequency is 27.1 per 1000 live births. Of these, the study found that 7.1 per 1000 presented with cardiovascular anomalies and 4.1 per 1000 presented with musculoskeletal anomalies [9]. Another relevant study found that there was an anomaly frequency rate of 41.2 per 1000 live births in Saudi Arabia, with 1.3 per 1000 presenting with gastrointestinal anomalies and 1.9 per 1000 presenting with neural tube anomalies [9]. A vast range of causal factors is at play here, with nutritional, genetic, environmental, economic and lifestyle variables often interacting with one another and medical treatments and infections also comprising potential causes [10]. Ultrasound examinations can be used to spot congenital anomalies in foetuses, including neural tube anomalies, cardiovascular anomalies, cleft lip and palate, or mal-developments in the limbs. Medical perinatal treatment of the child can be better planned when ultrasound examinations successfully identify anomalies and parents and families can be supported and prepared in anticipation of the issues that will arise in caring for the child [11]. Anomalous developments in the foetus's cranium, skeleton, chest, cardiovascular system, respiratory system, abdomen and limbs can often be identified via ultrasonography [12] and the procedure is not inexpensive, safe for the patient and non-invasive. Sonography techniques can be used to generate accurate pictures of the mother's uterus and the foetus that can be used to diagnose many anomalies and there are no major adverse effects on the mother or the foetus. Medical history has seen significant developments in the methods deployed to observe human anatomy and ultrasound technology has facilitated huge improvements in our capacity to understand both anatomy and the natural environment [13]. Foetal anomalies have been known of for longer than we have had access to ultrasound technology, with congenital anomalies affecting around 3-5% of the global population and the introduction of ultrasonography as a diagnostic procedure in obstetrics has significantly advanced physicians' understanding of them [14]. The health of the child, the coping abilities of mothers and families and the physical health of the mother can be enhanced when ultrasounds are used to accurately diagnose foetal anomalies because prenatal care plans can be initiated earlier on [15-16] and referrals to specialists can be made based on the obtainment of a comprehensive and detailed diagnosis. The problems in terms of mental distress, economic burden and physical health that can occur when congenital anomalies hamper a child's health and longevity are chronic and serious, affecting all parties involved, from family members to healthcare providers [17]. Therefore, it is of interest to determine the effectiveness of using ultrasound imaging to diagnose foetal anomalies among pregnant women from Najran, Saudi Arabia and to ascertain the relationships between the wome's health, their demographic characteristics and the presence and nature of any anomalies detected.

## Methods:

## Study design:

The present study adopted a retrospective cross-sectional design, using data from ultrasounds conducted in the Maternity and Childre's Hospital in Najran between January 2023 and January 2024.

## Sampling:

The sample comprised 100 women who had been pregnant for a minimum of 14 weeks and who had undergone obstetric ultrasound assessments. The sample was aged between 20 and 40. Each participant granted informed consent to partake in the study. Women who had a medical history of fetal anomalies in their former pregnancies and women, who did not consent to partake, were removed from the sample. Using calculations to determine a suitable sample size (whereby a confidence level of 95%+ was achieved and a 5% margin of error was the maximum allowable), the total sample of 100 individuals was established as being statistically representative. The study needed the sample to be sufficiently large so as to be representative of the general population and the researchers based their calculations on the fact that between 3% and 5% of the overall population has been found to present with foetal anomalies, so a sample of 100 women should enable the study to pick up on prevalence and the proportions of different types of anomalies on a relatively generalizable scale.

## Data collection:

Demographic and clinical data, pertaining to the wome's ages, the foetuses' gender, family history and pre-pregnancy health conditions on the part of the mother, any complications that had occurred in the pregnancy and parity, was collected by referring to patient records and by using a questionnaire specifically designed for the present study. Complying with professional protocols pertaining to the screening of foetal anomalies, ultrasound assessments were conducted by a registered sonographer on each woman in the sample, employing a high-resolution ultrasound machine. To analyse the anatomy of the foetuses and to spot anomalies accurately, a combination of 2D imaging methods and 3D imaging methods was deployed and the procedure was conducted in an appropriate clinical screening setting so that the women felt comfortable and safe. The procedure involved the methodical documentation of important anatomical observations and of the presence of foetal anomalies where they occurred, using a checklist to conduct detailed assessments of vital anatomical features, notably the brain, the spine, the urinary system, the heart and the gastrointestinal tract. The checklist enabled the recording of any fetal anomalies according to specific anatomical categories - i.e., central nervous system, gastrointestinal, musculoskeletal, genitourinary, or cardiac anomalies.

## Data analysis:

The data was subjected to descriptive statistical analysis in order to capture mean(s), standard deviation(s), frequency and distribution across the demographic and medical variables, using SPSS (version 26) and the study calculated the mean and standard deviations for gestational ages and the mothers' ages so that the distribution of those variables could be identified. To gain an overview of the distribution of demographic and medical variables, the study determined the frequency distribution for age of gestation, different types of foetal anomalies, parity, medical histories of pathology on the part of the mother and the age of the mothers. These are shown in the tables and figures below. To determine whether there are any significant correlations between maternal history of disease and the presence of any foetal anomalies, the study involved conducting a Chi-square test using SPSS. When a relationship had a p-value below 0.05 this was deemed to denote a significant correlation. The study ultimately sought to ascertain whether maternal history of diseases was associated with higher rates of foetal anomalies.

## Ethical consideration:

Fundamental research ethics best practices were complied with throughout the study, with the protection of the privacy, well-being and rights of the participants being maintained as inalienable principles. The study acquired full and informed consent from each participant. The identities of the participants were not disclosed in any written material or orally and data pertaining to the participants was fully anonymised and stored securely and discreetly.

## Results:

In terms of the point of gestation, there was a mean gestation age of 26.5 weeks, with the range across the sample being between 14 weeks and 40 weeks (SD = 4.2). In terms of the frequency distribution of trimesters, 48% of the participants (n = 48) were in the second trimester of gestation, 35% of them (n = 35) were in the third trimester and 17% of them (n = 17) were in the first trimester. Relatively valid representation of the three trimesters was therefore achieved (Table 1). In terms of mean age, the participants, who ranged from 20 years to 40 years old, had a mean age of 29.8 (SD = 4.6), with 62% of them (n = 62) being in the 25-35 age group. This age range is roughly speaking considered the prime childbearing age. 21% of the participants were below the age of 25 (n = 21) and 17% were over the age of 35 (n = 17) (Table 1). 42% of the participants were nulliparous (n = 42) and 58% were multiparous (n = 58%), indicating a relatively equal split between nulliparity and multiparity. Of the segment of women who were multiparous, those who had had one former pregnancy made up 43.1% (n = 25). Those who had had two former pregnancies accounted for 31% (n = 18) of the sample. Those who had had three or more former pregnancies account for 25.86% (n = 15). The distribution observed here is therefore relatively representative of the wider population (Table 1). Figure 1 - (see PDF) presents the frequency distribution and percentage distribution of the five forms of anomaly that were observed in the present study of ultrasound assessments of 100 pregnant women in Najran, Saudi Arabia. The anomalies identified included five types: (i) cardiac, musculoskeletal, central nervous system gastrointestinal and genitourinary. 34% of the sample presented with a cardiac anomaly. 22% of the sample had fetuses with some form of musculoskeletal anomaly. 20% had foetuses with central nervous system anomalies. 15% consisted of fetuses with gastrointestinal anomalies and 9% of the sample comprised fetuses with genitourinary anomalies. 30% of the sample reported medical histories of disease of some kind (n= 30), with the frequency distribution of types of disease as follows: 40% of those with existing diseases had diabetes mellitus (n = 12); 27% of this sub-sample had hypertension (n = 8); 20% of them had some form of thyroid disorder (n = 6); and 13% of them had some form of autoimmune disorder (n = 4). A significant portion of the sample, therefore, had some form of existing health issue that can increase the risk of the onset of fetal anomalies, with the distribution showing diabetes mellitus, known to increase the risk of anomalies occurring, to be the most prevalent condition suffered by the women sampled (Table 2) and (Table 3). Regarding association between mother pathology and congenital anomalies we found that there is a significant correlation between maternal diabetes mellitus and the presence of fetal congenital anomalies (Chi-square value = 7.50, p = 0.006). However there is no a statistically significant correlation between hypertension, thyroid disorders and autoimmune disorders and fetal congenital anomalies, as indicated by Chi-square value of (0.00 and a p-value of 1.000), (2.00, p = 0.157) and (0.67, p = 0.414) respectively (Table 4).

## Discussion:

The presence of anomalies in the local population of Najran, Saudi Arabia and the distribution of different forms of anomaly, have been identified in this study of pregnant women and their foetuses. The study used ultrasound imaging to identify anomalies in the women who were scanned in the Maternal and Child Hospital in Najran and demographic and medical history data was collected pertaining to the pregnant women. The study has explored the possible relationships between key demographic/medical variables (e.g., stage of gestation, age of mother, parity, presence of pre-pregnancy diseases in the mother) and the development of foetal anomalies. In terms of confirming the clinical utility of conducting ultrasound examinations during different points of gestation, the study found that the bulk of the women were in their second trimester (48% of them), with the mean age of gestation being 26.5 weeks. This aligns with the existing knowledge we have that conducting ultrasound examinations during trimester two can help healthcare professionals to identify anomalies in the foetus. When anomalies are spotted at this stage, there is better opportunity to medically intervene and/or initiate measures to support parents in the pre-natal phase [14]. The gestational age distribution of the sample here is thus representative of commonplace practices in terms of pre-natal care, with many anomalies being identified via examinations conducted at the 18-20 weeks stage [18]. The fact that some of the examinations detected anomalies in our sample confirms that ultrasound assessments should be conducted in the second trimester as a standardised practice.

The descriptive statistics pertaining to the women's ages produced results that are consistent with data on the wider population, with 62% of the sample being aged 25-35. The mean age was 29.8 years old. International data and analysis has shown that the age of the pregnant woman can significantly affect the risk of the development of foetal anomalies. Especially when abnormal chromosomal phenomena occur, but more generally, women who are pregnant who are either under 25 or over 35 have an increased risk of having a foetus with a congenital anomaly [19]. The predominance of mothers aged within what is considered the safe age range for childbearing indicates that anomaly prevalence could be worse if a greater proportion of the women were outside of that age range, but regular and detailed scans of all pregnant women are necessary regardless. The descriptive analysis of the data pertaining to the women's parity showed that 58% of the sample was comprised of multiparous women. Much of the research to date has found that parity has a strong link with the nature of pregnancy outcomes and difficulties, with nulliparous and multiparous women often encountering distinct issues [20]. 43.1% of the sample was comprised of multiparous women who had undergone a single former pregnancy and this has been linked to inexperience and additional support needs as compared to multiparous women who have undergone multiple former pregnancies. Some studies have found that when women are less experienced in terms of going through a pregnancy, certain risk factors can be increased, so a more thorough support plan should be targeted at pregnant women deemed by their healthcare workers to be inexperienced [21]. Our analysis of the prevalence of different types of foetal anomaly found that 34% of the sample had some form of cardiac anomaly and 22% had some form of musculoskeletal anomaly. Other studies have found similar rates of prevalence of these types of anomaly [22]. Having an accurate comprehension of the frequency of specific types of anomaly (among healthcare professionals and at the institutional level) is important when it comes to developing practices that will enable accurate diagnosis during pregnancy.

Cardiac anomalies are a major cause of chronic health issues in the child and a well-known driver of infant death, so ensuring that cardiac anomalies are comprehensively scanned for in all pregnant women is important. In terms of the relationship between a history of disease in the mother and the occurrence of foetal anomalies, this study found that 30% of the sample had some form of pre-pregnancy disease, with 40% of this sub-sample having diabetes mellitus, 27% having hypertension and 20% having some form of thyroid disorder. However, we found that there is a statistically significant correlation between maternal diabetes mellitus and the presence of fetal congenital anomalies (Chi-square value = 7.50, p = 0.006). However there is no a statistically significant correlation between hypertension, thyroid disorders and autoimmune disorders and fetal congenital anomalies, as indicated by Chi-square value of (0.00 and a p-value of 1.000),(2.00, p = 0.157) and (0.67, p = 0.414) respectively. The evidence to date is strongly indicative of the fact that foetal anomalies are more likely to occur in mothers who have certain pre-pregnancy health conditions, with women who have diabetes being at significantly greater risk of having foetuses with cardiac or neural tube anomalies [23]. Thorough assessment of the mother's existing health status and monitoring of any diseases the mother has are imperative in militating against foetal anomalies. Based on our study finding it is important for health professional to integrate routine evaluation for pregnant ladies health in prenatal care protocol also they will be education program for pregnant women about important of chronic health condtion on pregnancy outcome. Alqarawi *et al.* [24] report that lack of mother knowledge is within the challenge of having a child with congenital anomalies in Saudi Arabia.

## Conclusion:

In association between maternal pathology and fatal congenital anomalies in a sample of 100 women at Najran, Saudi Arabia is shown. Further studies with large population are needed to correlate between maternal pathologies and fatal congenital anomalies.

## Figures and Tables

**Table 1:** Frequency distribution of gestational stage, mother age and parity

**Gestational Stage**	**Frequency (n=100)**	**Percentage**
First trimester	17	17%
Second trimester	48	48%
Third trimester	35	35%
Mean gestational age of the foetuses 26.5 ± 4.2 weeks.		
Total	100	100%
Mother age (years		
Under 25	21	21%
25-35	62	62%
Over 35	17	17%
Mean age of mothers 29.8 ± 4.6 years.		
Total	100	100%
Parity Status		
Nulliparous	42	42%
Multiparous	58	58%
Total	100	100%
Multiparous women		
One previous pregnancy	25	43.10%
Two previous	18	31%
pregnancies		
Three or more	15	25.86%
previous pregnancies		
Total	58	100%

**Table 2:** Distribution of mother pathology

**Pathology Status of mothers**	**Frequency (n=100)**	**Percentage**
Pre-existing Pathology	30	30%
No pre-existing pathology	70	70%
Total	100	100%

**Table 3:** Frequency distribution of mother pathology

**Types mother pathology**	**Frequency (n=30)**	**Percentage**
diabetes mellitus	12	40%
hypertension	8	27%
thyroid disorders	6	20%
autoimmune disorders	4	13%
Total	30	100%

**Table 4:** Correlation between maternal pathology and fetal congenital anomalies

**Maternal pathology**	**Total n =30**	**Chi-square Value**	**p-value**	**Significance**
Diabetes Mellitus	12	7.5	0.006 < 0.05	Statistically significant
Hypertension	8	0	1.000> 0.05	Not Statistically significant
Thyroid Disorders	6	2	0.156> 0.05	Not significant
Autoimmune Disorders	4	0.67	0.414> 0.05	Not significant
